# Internet Addiction and Related Psychological Factors Among Children and Adolescents in China During the Coronavirus Disease 2019 (COVID-19) Epidemic

**DOI:** 10.3389/fpsyt.2020.00751

**Published:** 2020-09-02

**Authors:** Huixi Dong, Fangru Yang, Xiaozi Lu, Wei Hao

**Affiliations:** ^1^ Mental Health Institute of the Xiangya Hospital, National Clinical Research Center for Geriatric Disorders of Xiangya Hospital, Central South University, Changsha, China; ^2^ Qingdao Mental Health Center, Qingdao University, Qingdao, China; ^3^ Mental Health Institute of the Second Xiangya Hospital, Central South University, The China National Clinical Research Center for Mental Health Disorders, National Technology Institute of Psychiatry, Key Laboratory of Psychiatry and Mental Health of Hunan Province, Changsha, China

**Keywords:** Internet addiction (IA), children and adolescents, depression, anxiety, stress

## Abstract

**Background:**

The Coronavirus disease 2019 (COVID-19) is an infectious disease presenting a major threat to public health. This study aims to assess Internet use characteristics and objectively examine the potential psychological factors associated with Internet addiction (IA) during the COVID-19 epidemic.

**Methods:**

A cross-sectional, anonymized, self-reported survey was conducted among Chinese children and adolescents aged 6 to 18 years old. Participants completed questionnaires containing Young’s Internet Addiction Test (IAT) and the Depression, Anxiety, and Stress Scale (DASS-21), and questions regarding demographic information and Internet use characteristics.

**Results:**

A total of 2050 participants (mean age:12.34 ± 4.67 years old, female: 48.44%) were enrolled. Fifty-five (2.68%) participants met the criterion for addictive Internet use (IAT≥70), while 684 (33.37%) participants were classified as problematic Internet users (69≥IAT≥40). Internet usage had grown during the COVID-19 epidemic, including the frequency and duration of recreational Internet use, and the frequency of stay-up Internet use. A linear regression analysis showed female gender (β=-0.091, p<0.001), age (β=0.066, p=0.001), depression (β=0.257, p<0.001), and stress (β=0.323, p<0.001) were significantly correlated with the IAT total scores (R=0.539, R^2 =^ 0.291, p<0.001).

**Conclusions:**

We observed excessive Internet use among Chinese children and adolescents during the outbreak of COVID-19. Age, gender, depression, and stress were the potential key factors affecting IA. Extended family and professional support should be considered for vulnerable individuals during these unprecedented times.

## Introduction 

In December 2019, the Coronavirus disease 2019 (COVID-19) was first reported in Wuhan city, Hubei province, China (1). The COVID-19 is an extremely contagious disease with high infectivity, fast transmitting speed, susceptibility of all-age groups, and damage to public health. The emergency response and massive vigorous actions taken by the Chinese government have slowed down the epidemic. However, the Chinese people were facing enormous pressure and a grim challenge of prevention and control. The ministry of education of China issued notices in January, the extension of the opening of the spring semester in 2020, requiring all primary and secondary schools to delay school opening and students to stay at home (2) and learn *via* online courses (3). As an unprecedented, nationwide, even worldwide public health emergency, the epidemic of COVID-19 is bound to have a corresponding impact on the psychology and behaviors of school-age children and adolescents, who deserves more attention.

The rapid rise of the Internet age has popularized Internet use in China. More and more children and adolescents spend time on the Internet to study, play online games, shop, watch movies, use social media, and chat. These activities are often used to reduce stress and anxiety or to alleviate depressed mood. As there are 588 million Internet users in China (including 287 million teenagers) that account for 20% of all Internet users worldwide (4). Internet use in a reasonable way is beneficial, but excessive and uncontrolled Internet use may develop into Internet addiction (IA), which is defined as an individual’s inability to control his/her use of the Internet. IA is a serious public health problem in the world, especially in Asia (5). In China, the prevalence of IA has been reported as 2.4% to 10% (6, 7).

The COVID-19 outbreak is an excellent opportunity to study the association between stressful life events, its consequent psychological responses, and addictive behaviors. Previous studies have confirmed stress, depression, and anxiety are correlated with IA (8–12). We hypothesized that the increased level of stress, anxiety, and depression caused by the crises of the COVID-19 outbreak might change Internet use behaviors. Research data are needed to develop evidence-driven strategies to reduce adverse psychological impacts on Internet use. So this population-based epidemiological study screened, described, and compared the Internet addictive behaviors and identified risk factors of IA among school-aged children and adolescents in China in response to the outbreak.

## Methods

### Study Design

The popularization of Internet services and smartphones have enabled mental health professionals to provide online mental health investigation and assistance during the COVID-19 outbreak. As the Chinese Government recommended that the public to minimize face-to-face interaction, we conducted a cross-sectional study and electronically invited participants continuity through the WeChat-based ‘SurveyStar’ online platform (Changsha Ranxing Science and Technology, Shanghai, China). The ‘SurveyStar’ is a website providing online survey and statistical analysis functions equivalent to Amazon Mechanical Turk, from February 19, 2020 to March 15, 2020. The original sample was obtained from Hunan province, Shandong province, and Inner Mongolia Autonomous Region, based on geographic location (central, east, and north of China). There were 1019 confirmed COVID-19 cases and 4 deaths in Hunan province, 775 confirmed cases and 7 deaths in Shandong province, and 117 confirmed cases and 1 death in Inner Mongolia Autonomous Region (up to April 2, 2020). Official online accounts of hospitals released the survey.

### Samples

School-age children and adolescents from these three areas were recruited through the anonymous online questionnaire, designed by the first author and reviewed by senior psychiatrists. The admission criteria: (1) 6-18 years old, male or female, (2) voluntary participation in this survey, (3) ability to complete the online survey or with the help of parents, (4) being a student in primary or middle school, (5) signing informed consent online. The exclusion criteria: diagnosed with cognitive impairment, organic brain diseases, or severe mental disorders. The research protocol and consent procedures were approved by the Human Ethics Committee of the Xiangya Hospital of Central South University (the ethical approval number: 202003034), which conformed to the principles embodied in the Declaration of Helsinki.

### Measures

We obtained demographic data, including gender, date of birth, whether they were an only child or not, current study section, growth environment in the past three years, family income in the past one year, and educational level of participants’ mother and father. Participants were asked if they had ever been diagnosed with cognitive impairment, organic brain diseases, or mental disorders (schizophrenia, depression, bipolar disorders, obsessive-compulsive disorder, anxiety disorder, or substance use disorders) in their lifetime. If the answer was yes, they were excluded from the study.

We included several measures of Internet use, the primary electronic device of Internet use (smartphones or tablets, Internet protocol television (IPTV), computers, others), the frequency of recreational use of electronic devices (several times per day, once per day, 4-6 times per week, 1-3 times per week, no use); the duration of recreational use of electronic devices (>6 hours per day, 4-6 hours per day, 2-4 hours per day, <2 hours per day, no use); the frequency of recreational use of electronic devices after 00:00/week (>4 times per week, 3 times per week, twice per week, once per week, no use); the frequency of recreational use of electronic devices overnight/week (>4 times per week, 3 times per week, twice per week, once per week, no use); the degree of addiction to electronic devices (self-rating, 0-100 scores) during and before the epidemic of COVID-19.

The Chinese version of Young’s Internet Addiction Test (IAT) was used to screen symptoms of IA. It is a self-rated test with 20 items, and each item is scored on a scale of 1-5. According to Young et al.’s criteria (13), participants whose IAT total scores 70 or above were classified as addictive Internet users (AIU) who had encountered significant life problems due to Internet use. Participants with an IAT total score of 40-69 were classified as problematic Internet users (PIU) who had encountered general life problems due to Internet use. Participants with an IAT score of 39 or below were classified as normal Internet users (NIU), who only had some or no problems controlling Internet use. Adequate reliability of IAT in Chinese languages compared with English (Cronbach alpha≥0.82) (14, 15).

The mental state was assessed using the Chinese version of Depression, Anxiety, and Stress Scale (DASS-21). DASS-21 is a screening tool to measure depression, anxiety, and stress in the reference period of “past one week.” Questions 3, 5, 10, 13, 16, 17, and 21 formed the depression subscale. The total depression subscale score was divided into normal (0–9), mild to moderate depression (13–27), and severe depression (28–42). Questions 2, 4, 7, 9, 15, 19, and 20 formed the anxiety subscale. The total anxiety subscale score was divided into normal (0–7), mild to moderate anxiety (8–14), severe anxiety (15-42). Questions 1, 6, 8, 11, 12, 14, and 18 formed the stress subscale. The total stress subscale score was divided into normal (0–10), mild to moderate stress (11–25), severe stress (26–2). The Chinese version of DASS-21 has been demonstrated to be a good psychometric screening tool with good validity and reliability in the Chinese population (16), even in adolescents (17, 18) and research during the severe acute respiratory syndrome (SARS) outbreak (19).

### Statistical Analysis

Data online was exported in.sav format. Simple descriptive statistics were expressed as ‘mean and standard deviation’ for continuous variables, ‘frequency and percentage’ for categorical variables. Differences in demographics, Internet use, and DASS-21 variables among groups were tested using t-test, ANOVA test, Pearson’s χ^2^ test, or Fisher’s exact test. The binary logistic regression with the ‘enter’ method was used to evaluate if factors significant in univariate analysis were strongly associated with AIU and PIU. We also used linear regression analysis to investigate the associations between demographic characteristics, the subscales of the DASS-21 (depression, anxiety, and stress) and IAT total scores. The odds ratios (OR), corresponding to 95% of confidence intervals (CI), standardized coefficient β values were generated for each variable. All tests were two-tailed, with a significance level of p<0.05. To adjust for multiple post-hoc test the significance level was set to p<0.014 (α’=(2*α)/[n*(n-1)+1], n=3). Statistical analysis was performed using SPSS Statistic 21.0 (IBM SPSS Statistics, New York, United States).

## Results

Of the 2270 surveys administered, 39 surveys were interrupted because they did not agree to participate in this research, and 181 surveys were removed, belonging to respondents who were less than 6 years old or more than 18 years old. The final participants were 2050 children and adolescents aged 6–18 years old from three different areas (660 from Hunan province, 651 from Shandong province, and 739 from Inner Mongolia Autonomous Region). The mean age of participants was 12.34 (SD: 4.67) years old (males, 12.44 (SD: 5.45) years old, n=1057; girls, 12.24 (SD: 3.661) years old, n=993).

### Incidence of Addictive, Problematic Internet Use and Demographic Characteristics

The mean score of total IAT was 36.83 (SD, 13.80); Male, 37.86 (SD, 14.53); Female, 35.72 (SD, 12.89)). Based on total IAT scores, 2.68% (Male: 3.50%; Female: 1.81%) and 33.37% (Male, 35.10%; Female, 31.52%) of the participants were classified as addicted and excessive Internet users, respectively. The mean IAT score was 78.96 (SD,7.303) in the AIU, 49.38 (SD, 7.603) in the PIU, and 28.51 (SD, 6.275) in the NIU. We found age, gender, and education status were significantly different among AIU, PIU, and NIU (*p*<0.001). There were no significant differences in the rate of the only child, residence, annual family income, or mother’s or father’s education level in the three groups (see **Table 1**).

**Table 1:** Comparison of demographic characteristics among subsamples of addictive, problematic, and normal Internet use.

	Addictive Internet Users (n=55)	Problematic Internet Users (n=684)	Normal Internet Users (n=1311)	*χ2*	*p*
Age, *mean (SE), n (%)*	12.95 (3.297)	12.78 (3.791)	12.09 (5.101)		
6˜9	7 (12.7)	118 (17.3)	295 (22.5)	38.692	<0.001
10˜14	29 (52.7)	318 (46.5)	706 (53.9)		
15˜18	19 (34.5)	248 (36.3)	310 (23.6)		
Gender, *n (%)*					
Male	37 (67.3)	371 (54.2)	649 (49.5)	994.610	<0.001
Female	18 (32.7)	313 (45.8)	662 (50.5)		
The only child, *n (%)*				2.439	0.295
Yes	29 (52.7)	322 (47.1)	663 (50.6)		
No	26 (47.3)	362 (52.9)	648 (49.4)		
Education status, *n (%)*				70.601	<0.001
Primary school	14 (25.5)	207 (30.3)	624 (47.6)		
Junior middle school	30 (54.5)	296 (43.3)	479 (36.5)		
Senior middle/Technical secondary school	11 (20.0)	181 (26.5)	208 (15.9)		
Residence, *n (%)*				7.722	0.259
First- and second-tier cities	8 (14.5)	121 (17.7)	231 (17.6)		
Third- and fourth-tier cities	36 (65.5)	363 (53.1)	758 (57.8)		
Villages and towns	9 (16.4)	151 (22.1)	247 (18.8)		
Rural area	2 (3.6)	49 (7.2)	75 (5.7)		
Annual family income (RMB yuan), *n (%)*				4.123	0.660
<5000	19 (34.5)	174 (25.4)	375 (28.6)		
5000-10000	23 (41.8)	332 (48.5)	602 (45.9)		
10000-30000	9 (16.4)	132 (19.3)	239 (18.2)		
>30000	4 (7.3)	46 (6.7)	95 (7.2)		
Mother’s Education Level, *n (%)*				9.294	0.158
Primary school or below	12 (21.8)	70 (10.2)	156 (11.9)		
Junior middle school	13 (23.6)	172 (25.1)	355 (27.1)		
Senior middle/Technical secondary school	10 (18.2)	183 (26.8)	317 (24.2)		
College/Bachelor degree or above	20 (36.4)	259 (37.9)	483 (36.8)		
Father’s Education Level, *n (%)*				9.105	0.168
Primary school or below	8 (14.5)	57 (8.3)	96 (7.3)		
Junior middle school	15 (27.3)	168 (24.6)	384 (29.3)		
Senior middle/Technical secondary school	12 (21.8)	183 (26.8)	318 (24.3)		
College/Bachelor degree or above	20 (36.4)	276 (40.4)	513 (39.1)		

Categorical variables were compared using Chi-square test.

### Electronic Device Use During and Before the Epidemic of COVID-19

**Figure 1:**
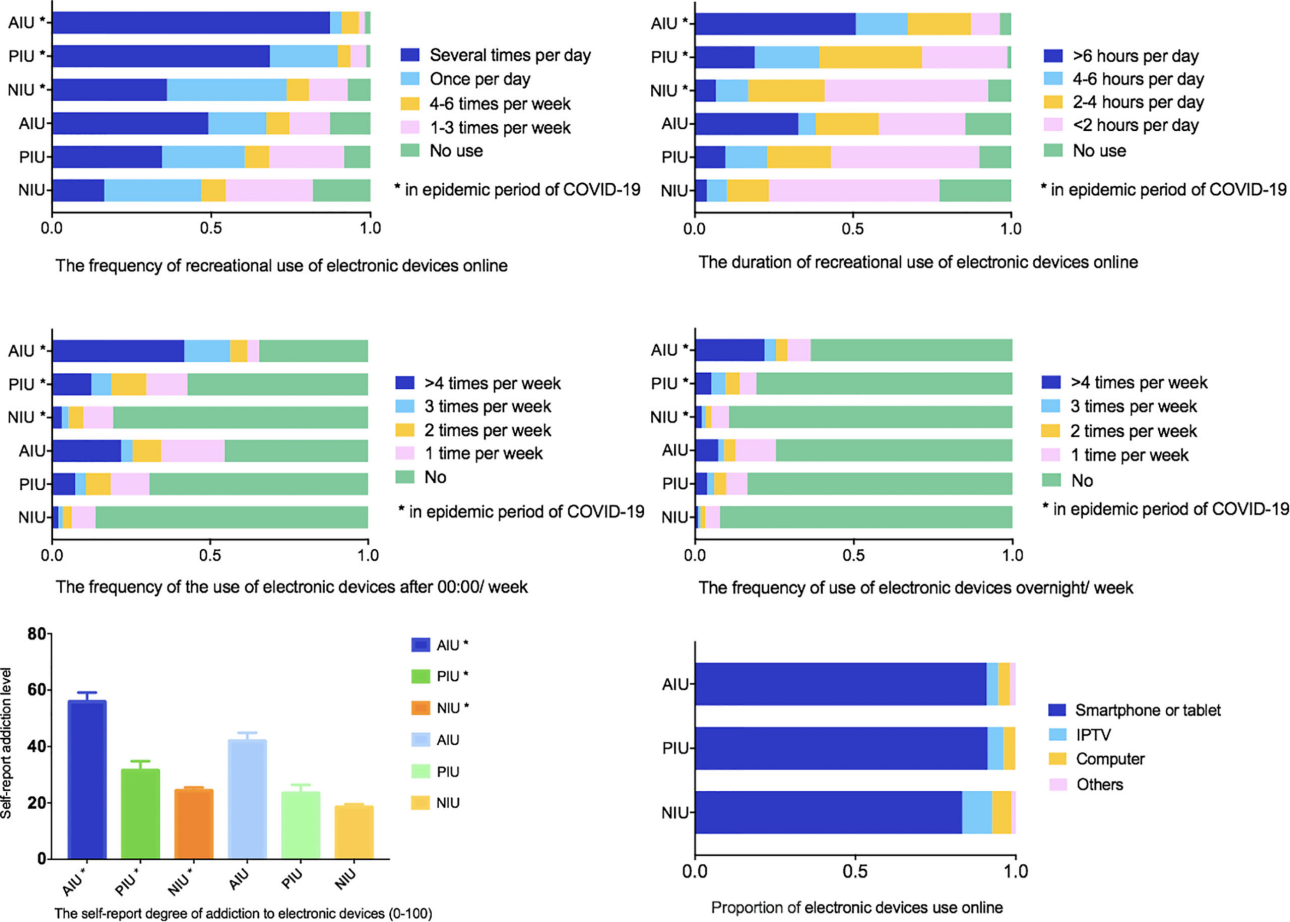
Characteristic of recreational use of electronic devices online during and before the epidemic of COVID-19.

Smartphones or tablets were the primary electronic devices (AIU, 90.91%; PIU, 91.23%; NIU, 83.30%), followed by IPTV (AIU, 3.64%; PIU, 4.97%; NIU, 9.31%), computers (AIU, 3.64%; PIU, 3.51%; NIU, 5.95%) and other devices (AIU,1.82%; PIU, 2.92%; NIU, 1.45%%). For AIU, PIU and NIU groups, the frequency of recreational use of electronic devices online, the duration of recreational use of electronic devices online, the frequency of the use of electronic devices after 00:00/week, and the degree of addiction to electronic devices (self-rating, 0-100 scores) during the epidemic of COVID-19 were all increased than before. However, the frequency of use of electronic devices overnight/week increased only in NIU, but not in AIU and PIU (**Table 2**, **Figure 1**).

**Table 2:** Recreational use of electronic devices among subsamples of addictive, problematic, and normal Internet use during and before the epidemic of COVID-19.

Recreational use of electronic devices	Addictive Internet Users(n = 55)		Problematic Internet Users (n = 684)		Normal Internet Users (n = 1311)		Comparison
	During the epidemic	Before theepidemic	During the epidemic	Before the epidemic	During the epidemic	Before the epidemic	
Frequency, *n (%)*							A*** P*** N***
several times per day	48 (87.27)	27 (49.09)	468 (68.42)	236 (34.50)	473 (36.08)	215 (16.40)
once per day	2 (3.64)	10 (18.18)	146 (21.35)	178 (26.02)	493 (37.60)	400 (30.51)
4-6 times per week	3 (5.45)	4 (7.27)	27 (3.95)	53 (7.75)	92 (7.02)	100 (7.63)
1-3 times per week	1 (1.82)	7 (12.73)	34 (4.97)	160 (23.39)	159 (12.13)	358 (27.31)
no use	1 (1.82)	7 (12.73)	9 (1.32)	57 (8.33)	94 (7.40)	238 (18.15)
Duration per day, *n (%)*							A** P*** N***
>6 hours	28 (50.91)	18 (32.73)	129 (18.86)	66 (9.65)	87 (6.64)	49 (3.74)
4-6 hours (including 6 hours)	9 (16.36)	3 (5.45)	140 (20.47)	90 (13.16)	134 (10.22)	84 (6.41)
2-4 hours (including 4 hours)	11 (20.00)	11 (20.00)	222 (32.46)	138 (20.18)	317 (24.18)	173 (13.20)
<2 hours	5 (9.09)	15 (27.27)	184 (26.90)	321 (46.93)	676 (51.56)	707 (53.93)
no use	2 (3.64)	8 (14.55)	9 (1.32)	69 (10.09)	97 (7.40)	298 (22.73)
Frequency of use after 00:00, *n (%)*							A** P** N**
>4 times per week	23 (41.82)	12 (21.82)	85 (12.43)	50 (7.31)	40 (3.05)	26 (1.98)
3 times per week	8 (14.55)	2 (3.64)	43 (6.29)	23 (3.36)	28 (2.14)	18 (1.37)
twice per week	3 (5.45)	5 (9.09)	76 (11.11)	54 (7.89)	62 (4.73)	37 (2.82)
once per week	2 (3.64)	11 (20.00)	89 (13.01)	83 (12.13)	122 (9.31)	98 (7.48)
no	19 (34.55)	25 (45.45)	391 (57.16)	474 (69.30)	1059 (80.78)	1132 (86.35)	
Frequency of use overnight, *n (%)*							N*
>4 times per week	12 (21.82)	4 (7.27)	35 (5.12)	26 (3.80)	27 (2.06)	12 (0.92)	
3 times per week	2 (3.64)	1 (1.82)	31 (4.53)	15 (2.19)	17 (1.30)	10 (0.76)	
twice per week	2 (3.64)	2 (3.64)	30 (4.39)	26 (3.80)	23 (1.75)	20 (1.53)	
once per week	4 (7.27)	7 (12.73)	36 (5.26)	45 (6.58)	72 (5.49)	60 (4.57)	
no	35 (63.64)	41 (74.55)	552 (80.70)	572 (83.63)	1172 (89.40)	1209 (92.22)	
Degree of addiction to electronic devices/mean *(SE)*	55.92 (3.21)	41.97 (2.90)	31.54 (3.27)	23.44 (2.95)	24.38 (1.10)	18.53 (0.99)	A*** P*** N***

A: Difference in addictive Internet users during and before the epidemic of COVID-19.

P: Difference in problematic Internet users during and before the epidemic of COVID-19.

N: Difference in average Internet users during and before the epidemic of COVID-19.

*P < 0.05,**P < 0.01, ***P < 0.001.

### Prevalence of Depression, Anxiety, and Stress During the Epidemic of COVID-19

The prevalence of depression, anxiety, and stress were found to be 17.66% (n=362), 15.54% (n=298), and 7.07% (n=145), respectively. The prevalence of different levels of depression, anxiety, and stress in the past one week differed significantly among the three groups (*p*<0.001). Post-hoc analyses respectively showed differences between the AIU and PIU, AIU and NIU, as well as the PIU and NIU in **Table 3** (*p*<0.014).

**Table 3:** Prevalence of depression, anxiety, and stress among subsamples of addictive, problematic, and normal Internet users.

	Addictive Internet Users (n = 55)	Problematic Internet Users (n = 684)	NormalInternet Users (n = 1311)	*χ2*	*p*	Paired comparisons
Depression, *n (%)*				331.00	<0.001	1*;2*;3*
Severe	15 (27.27)	49 (7.16)	7 (0.53)			
Mild to moderate	17 (30.91)	182 (26.61)	92 (7.02.)			
No	23 (41.82)	453 (66.23)	1212 (92.45)			
Anxiety, n (%)				267.66	<0.001	1*;2*;3*
Severe	15 (27.27)	52 (7.60)	6 (0.46)			
Mild to moderate	9 (16.36)	138 (20.18)	78 (5.95)			
No	31 (56.36)	494 (72.22)	1227 (93.59)			
Stress, *n (%)*				207.58	<0.001	1*;2*;3*
Severe	11 (20.00)	32 (4.68)	3 (0.23)			
Mild to moderate	9 (16.36)	67 (9.80)	23 (1.75)			
No	35 (63.64)	585 (85.53)	1285 (98.02)			

α’=(2*α)/[3*(3-1)+1]=0.014

1: Addictive Internet Users vs Problematic Internet Users

2: Addictive Internet Users vs Normal Internet Users

3: Problematic Internet Users vs Normal Internet Users

*p < 0.014.

### Associations Between Demographic Characteristics, Depression, Anxiety, Stress, and Addictive, Problematic Internet Use

We used two statistical models to explore the factors which may contribute to Internet addiction. As shown in **Table 4**, the binary logistic regression analysis shows that male gender (OR=1.491, *p*<0.001), age (OR=1.073, *p*<0.001), severe depression (OR=3.672, *p*=0.003), mild to moderate depression (OR=2.881, *p*<0.001), mild to moderate anxiety (OR=1.831, *p*=0.001), severe stress (OR=4.500, *p*=0.020), and mild to moderate stress (OR=2.508, *p*=0.009) were significantly associated with AIU and PIU. No significant association was observed between addictive, problematic Internet use and severe anxiety (*p*=0.087). Furthermore, the linear regression analysis (**Table 5**) shows female gender (β=-0.091, *p*<0.001), age (β=0.066, *p*=0.001), depression (β=0.257, *p*<0.001), and stress (β=0.323, *p*<0.001) were significantly correlated with the IAT total score (R=0.539, R^2 =^ 0.291, *p*<0.001).

**Table 4:** Logistic regression analysis of risk factors of addictive, problematic Internet use.

Variables	OR (95% CI)	*P*
Male	1.491 (1.220~1.822)	<0.001
Age	1.073 (1.037~1.111)	<0.001
Depression		
Severe	3.672 (1.579~8.543)	0.003
Mild to moderate	2.881 (2.093~3.965)	<0.001
Anxiety		
Severe	2.095 (0.899~4.884)	0.087
Mild to moderate	1.831 (1.285~2.609)	0.001
Stress		
Severe	4.500 (1.266~15.995)	0.020
Mild to moderate	2.058 (1.198~3.534)	0.009

OR, odds ratio; CI, confidence interval. *Analyzed by multiple logistic regression model.

**Table 5:** Linear regression of the relationships between research variables and IAT total score.

Variables	Standardized coefficient β	*p*
Female	-0.091	<0.001
Age	0.066	0.001
Depression	0.257	<0.001
Anxiety	-0.048	0.168
Stress	0.323	<0.001

## Discussion

### Prevalence of AUI and PUI Among Chinese Children and Adolescents

To the best of our knowledge, there is limited information available on an overview study of IA and related risk factors of children and adolescents in a particular situation of stress and isolated condition. In the present study, 2.68% and 33.37% of the participants were classified as addicted and possibly addicted to the Internet. Several studies using the same criteria by the IAT reported with 1.2%~6.2% addicted Internet users and 12.5%~46.0% problematic Internet users in China mainland, Hong Kong, Japan, South Korea, Malaysia, Philippines, Nigeria, and Greece (7, 20, 21). We found the prevalence was relatively higher than those reported earlier in China (2.2%/17.1%). The possible reason is that more students spent time in Internet use during this investigation. The fear resulting from the COVID-19 disease, and the consequences of lockdown, depression, and stress have been mounting affecting individuals’ behaviors. It may also be explained by methodological differences (cross-sectional versus longitudinal study designs), and different statistical approaches (correlations versus time-lag models).

### Age and IA

The results also showed that IA grew with age. Our research had a young group aged from 6 to 9 years old, and the incidence of AUI and PUI was 1.67% and 28.10%, respectively. Until now, most studies focused on the population of teenagers and young adults on Internet use (22). However, the onset use of the Internet in China has become earlier. The research report on the situation of Internet use among children and adolescents in China pointed out that the daily electronic devices (followed by mobile phones and tablets, followed by computers and televisions) exposure rate of Chinese children over 3 years old reached 92% (4). The average days of Internet use every week in children aged 3-8 years old in China are 3.7 (4). IA, just as Internet gaming disorder and gambling disorder, is a kind of addictive behavior without psychoactive substances. Behavioral addiction is generally developed incrementally and characterized by a change from fun, through losing control, to obsession (22). Internet use has its popularity and accessibility, so we should start to prevent IA in children according to the findings of this study.

### Gender and IA

Gender difference is an essential issue in terms of IA. The proportion of males in AUI was much higher than that of females, which is consistent with previous studies (21, 23, 24). Boys who recreationally use the Internet prefer massive multiplayer online role-playing games (MMORPGs) (25) and violent games (26). In contrast, girls’ online activities are mostly focused on playing time-killing games, socializing, texting, and online shopping, but less violent games (27–30). Girls present with shorter durations of online gaming and shorter online screen times (31). The possible reason is girls are better in self-control and emotional regulation and physically and psychologically mature earlier, which can reduce pathological Internet use, especially when negative events occur (32, 33).

### Internet Use and Mental State During the Epidemic of COVID-19

This study discovered that the frequency and duration of recreational electronic devices use, the frequency of electronic devices use after 00:00, and the self-score of addiction to electronic products were all significantly higher than those before the epidemic in all the groups. It is worth noting that the frequency of use overnight/week in NIU was higher than that before the epidemic, which might indicate risks in developing IA. Considerable attention should be paid to all children and adolescents, including those who have not yet reached the standard of IA.

Our data showed that a number of participants experienced significant depression, anxiety, and stress during the outbreak. In this particular period, due to the suspension of schools, the closure of living environments, the reduction of outdoor activities, and the increase of epidemic pressure, the mental health of school-age children and adolescents were threatened. The uncertainty and potentially adverse effect of a loss of academic progression could have a harmful impact. Furthermore, children and adolescents were not psychologically independent and still in the stage of psychological development, and also faced the challenge of massive online courses. Although guidance and handbooks for mental health care for Chinese people were posted and free online or telephone psychological counseling was widely promoted during the COVID-19 outbreak, there was little relative professional psychological assistance for children and adolescents. However, worth noting that we only assessed the psychological status once in the early phase of the outbreak. Given the ongoing pandemic, further studies on mental health over time and confirmation of psychological status development are needed.

Our study confirmed the role of depression and stress in IA in response to this outbreak, and these findings were inconsistent with the previous studies (8, 34–36). It is well-known that adverse experiences are associated with higher depression and stress levels (37). Internet is entertaining and easy to access, which may be a common way for children and adolescents to release emotions and stress and escape from reality (38). The Internet, especially online games, can stimulate individuals to have a sense of energy and autonomy and enhance self-esteem. Excessive users will be more focused on Internet and less interested in real life (39). Future studies are needed to assess whether expanding online consulting, particularly during the outbreak, effectively reduces depression and stress among children and adolescents, and indirectly prevents the development of IA.

### Limitations

Several limitations should be noted in the present study. Firstly, the cross-sectional survey does not allow a conclusion to be drawn from the risk factors to IA among children and adolescents, so the results should be interpreted carefully. Secondly, IA lacks definite diagnostic criteria and is not determined by psychiatrists internationally. Young’s Internet Addiction Test reflects mainly the DSM-IV’s criteria of addiction, and the results may differ if other assessments were used. Thirdly, this study was based on the subjects’ self-report, and pre-epidemic assessment may lead to memory bias. At last, as a response to the COVID-19 pandemic, the Chinese government has introduced steps of spatial distancing and “staying at home” to curb its spread and impact. So the sample was taken by a convenient sampling method to investigate mental health status and Internet use characteristics by online questionnaires. Despite these limitations, our findings still have important implications for research and intervention.

### Conclusion

This study is a timely and large sample size investigation of Internet use in the representative areas of China in the COVID-19 outbreak. The findings provide substantial evidence of excessive Internet use among Chinese children and adolescents during this outbreak. Internet use is mainly influenced by the COVID-19 epidemic in various ways, including frequency and duration of recreational Internet use, and the rate of stay-up use. This study also represents a preliminary step towards understanding the relationship between IA and potentially related factors (male gender, age, depression, and stress). Therefore, it is necessary to provide preventive measures and strengthen education on IA for children and adolescents in countries experiencing or recovering from the epidemic. Further investigation is justified into whether reducing depression and stress can prevent IA among children and adolescents. Nevertheless, because of the cross-sectional design of this study, large-scale prospective studies are warranted to confirm these associations.

## Data Availability Statement

The raw data supporting the conclusions of this article will be made available by the authors, without undue reservation.

## Ethics Statement

The studies involving human participants were reviewed and approved by The research protocol and consent procedures were approved by the Human Ethics Committee of the Xiangya Hospital of Central South University (the ethical approval number: 202003034). Written informed consent to participate in this study was provided by the participants’ legal guardian/next of kin. Written informed consent was obtained from the individual(s), and minor(s)’ legal guardian/next of kin, for the publication of any potentially identifiable images or data included in this article.

## Author Contributions

HD: writing of article, acquisition of data, and analysis/interpretation. FY: contributed to the conception and design, analysis/interpretation, and final approval for publication. XL: contributed to acquisition of data. WH: contributed to the analysis/interpretation, and final approval for publication.

## Funding

This study was supported by grants from the Disciplinary Construction Research Grant of Xiangya Hospital (FY, PI), the Key Program of the National Natural Science of China (81130020, WH, PI), and the National 973 Program (2015CB553500, WH, PI).

## Conflict of Interest

The authors declare that the research was conducted in the absence of any commercial or financial relationships that could be construed as a potential conflict of interest.
